# Supplementary material for: Girgentana’s Goat Milk Microbiota Investigated in an Organic Farm During Dry Season
Source: Animals (Basel). 2025 Oct 30;15(21):3149. doi: 10.3390/ani15213149 (PMC12607308; doi:10.3390/ani15213149)
Supplement: Supplementary file 1 [file animals-15-03149-s001.zip › animals-3923432-supplementary.pdf]

animals

MDPI

Supplementary Materials

Table S1. Relative abundances of microbial taxa in goat milk samples (individual and bulk). The seven most prevalent taxa are reported at each taxonomic level (Phylum, Class, Order, Family, Genus), while all remaining taxa are grouped under the category 'Others'.

Taxonomic level	Individual milk	Bulk milk
**Phylum**		
Firmicutes	51.27%	51.49%
Proteobacteria	27.37%	24.21%
Actinobacteria	20.80%	24.14%
Cyanobacteria/Chloroplast	0.25%	0.14%
Tenericutes	0.23%	0.00%
Bacteroidetes	0.07%	0.02%
**Class**		
Bacilli	51.20%	51.01%
Actinobacteria	20.80%	24.14%
Gammaproteobacteria	14.33%	14.78%
Alphaproteobacteria	9.33%	6.01%
Betaproteobacteria	3.71%	3.42%
Chloroplast	0.25%	0.14%
Mollicutes	0.23%	0.00%
Others	0.15%	0.51%
**Order**		
Lactobacillales	51.27%	51.01%
Bifidobacteriales	20.69%	24.14%
Orbales	11.01%	11.80%
Rhizobiales	5.30%	1.30%
Rhodospirillales	4.03%	4.71%
Neisseriales	3.71%	3.42%
Enterobacteriales	3.30%	2.96%
Others	0.67%	0.66%
**Family**		
Lactobacillaceae	53.91%	51.83%
Bifidobacteriaceae	21.96%	24.59%
Orbaceae	11.68%	12.02%
Acetobacteraceae	4.29%	4.80%
Neisseriaceae	3.95%	3.48%
Enterobacteriaceae	3.44%	3.02%
Spiroplasmataceae	0.27%	0.00%
Others	0.50%	0.26%
**Genus**		
Lactobacillus	58.59%	55.59%
Bifidobacterium	22.85%	26.28%
Gilliamella	10.85%	8.96%
Snodgrassella	4.28%	3.70%
Frischella	1.56%	3.79%
Escherichia/Shigella	0.36%	0.40%
Spiroplasma	0.28%	0.00%

 https://doi.org/10.3390/xxxxx

Others	1.22%	1.50%

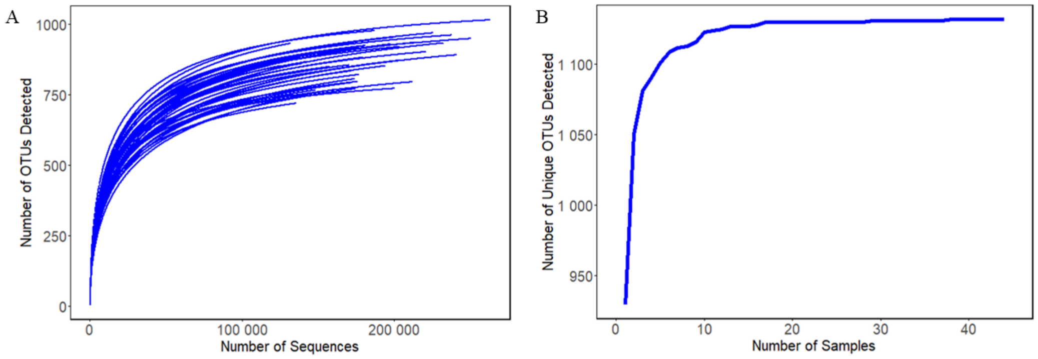

Figure S1. Sequence-based (A) and sample-based (B) rarefaction curves for the 44 samples of Girgentana's individual milk. The number of detected OTUs is on the y-axis, while the number of sequences and samples on the x-axis, respectively.

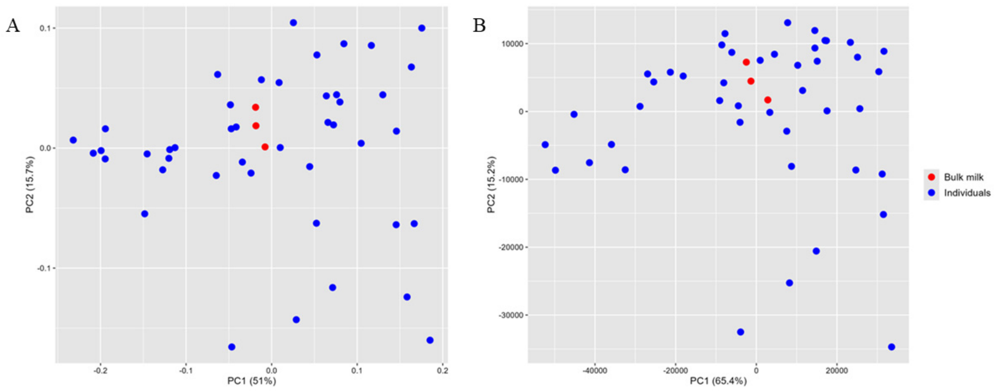

Figure S2. Principal Coordinates Analysis (PCoA) plots based on (A) Bray-Curtis and (B) Euclidean distance metrics. Each point represents a sample, colored by sample type (Individual vs Bulk milk).
